# Supplementary material for: Causal Relationship between Aging and Anorexia Nervosa: A White-Matter-Microstructure-Mediated Mendelian Randomization Analysis
Source: Biomedicines. 2024 Aug 16;12(8):1874. doi: 10.3390/biomedicines12081874 (PMC11351342; doi:10.3390/biomedicines12081874)
Supplement: Supplementary file 1 [file biomedicines-12-01874-s001.zip › Supplementary Figure/Figure S7/Single SNP plot of MR analysis for ubm-b-1733 to AN.pdf]

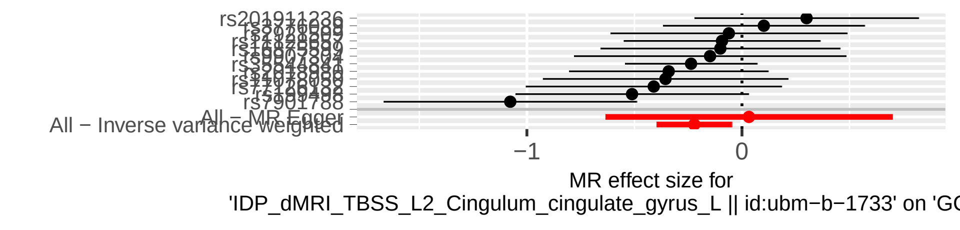

rs7901788
All − MR Egger
All − Inverse variance weighted
−1
0
MR effect size for
'IDP_dMRI_TBSS_L2_Cingulum_cingulate_gyrus_L || id:ubm−b−1733' on 'GC
